# Evaluation of Anticonvulsant Actions of Dibromophenyl Enaminones Using *In Vitro* and *In Vivo* Seizure Models

**DOI:** 10.1371/journal.pone.0099770

**Published:** 2014-06-19

**Authors:** Mohamed G. Qaddoumi, Kethireddy V. V. Ananthalakshmi, Oludotun A. Phillips, Ivan O. Edafiogho, Samuel B. Kombian

**Affiliations:** 1 Department of Pharmacology & Therapeutics, Faculty of Pharmacy, Kuwait University, Safat, Kuwait; 2 Department of Pharmaceutical Chemistry, Faculty of Pharmacy, Kuwait University, Safat, Kuwait; 3 Department of Pharmaceutical Sciences, School of Pharmacy, University of Saint Joseph, Hartford, Connecticut, United States of America; Dalhousie University, Canada

## Abstract

Epilepsy and other seizure disorders are not adequately managed with currently available drugs. We recently synthesized a series of dibromophenyl enaminones and demonstrated that AK6 and E249 were equipotent to previous analogs but more efficacious in suppressing neuronal excitation. Here we examined the actions of these lead compounds on *in vitro* and *in vivo* seizure models. *In vitro* seizures were induced in the hippocampal slice chemically (zero Mg^2+^ buffer and picrotoxin) and electrically using patterned high frequency stimulation (HFS) of afferents. *In vivo* seizures were induced in rats using the 6 Hz and the maximal electroshock models. AK6 (10 µM) and E249 (10 µM) depressed the amplitude of population spikes recorded in area CA1 of the hippocampus by −50.5±4.3% and −40.1±3.1% respectively, with partial recovery after washout. In the zero Mg^2+^ model, AK6 (10 µM) depressed multiple population spiking (mPS) by −59.3±6.9% and spontaneous bursts (SBs) by −65.9±7.2% and in the picrotoxin-model by −43.3±7.2% and −50.0±8.3%, respectively. Likewise, E249 (10 µM) depressed the zero-Mg^2+^-induced mPS by −48.8±9.5% and SBs by −55.8±15.5%, and in the picrotoxin model by −37.1±5.5% and −56.5±11.4%, respectively. They both suppressed post-HFS induced afterdischarges and SBs. AK6 and E249 dose-dependently protected rats in maximal electroshock and 6 Hz models of *in vivo* seizures after 30 min pretreatment. Their level of protection in both models was similar to that obtained with phenytoin Finally, while AK6 had no effect on locomotion in rats, phenytoin significantly decreased locomotion. AK6 and E249, suppressed *in vitro* and *in vivo* seizures to a similar extent. Their *in vivo* activities are comparable with but not superior to phenytoin. The most efficacious, AK6 produced no locomotor suppression while phenytoin did. Thus, AK6 and E249 may be excellent candidates for further investigation as potential agents for the treatment of epilepsy syndromes with possibly less CNS side effects.

## Introduction

Epilepsy is a chronic disorder of the central nervous system which is characterized by recurrent highly synchronized spontaneous discharges of large groups of neurons often of cortical origin [1], [2]. Current treatment is mainly by use of antiepileptic drugs (AEDs) most of which have side effects, such as somnolence, drowsiness and ataxia, which reduce the quality of life by interfering with certain activities of daily living of sufferers [3]–[7]. Furthermore, between 20% to 40% of sufferers are classified as treatment resistant as they do not respond to current medications [8]. The mechanisms of resistance to AEDs is currently the subject of intense investigations with calls for a radical rethink of antiepileptic therapy to include antiepileptogenic agents [9]–[11]. The ideal AED should, among other criteria [6], be anti-ictal, antiepileptogenic and/or disease modifying [12], [13]. Although it may be impossible to come up with one drug that does all these effects, the need to continue research on discovery of new AEDs that either alone or in combination meet these challenges is paramount [4], [14]. To this end, we and others have focused our efforts on the structural modification of the new enaminone pharmacophore in order to synthesize newer and more potent derivatives. To date, no member of the enaminone class of compound is as yet clinically available, although some of the new derivatives have demonstrated potential for anticonvulsant activity [15]–[19] on *in vitro* and *in vitro* models.

Several *in vivo* and *in vitro* models have been developed for the testing of new compounds for anticonvulsant activity and to study the abnormal synchronous activity of neurons in epilepsy [4], [14], [20]–[23]. Appropriate *in vitro* seizure models, chemically- and electrically-induced, are useful to provide initial screening of novel compounds. These models enable investigations on the mechanism(s) of action of potential anticonvulsant agents on network phenomena involved in neuronal synchronization and seizures [24], [25]. These models use brain slices with low seizure threshold, such as the hippocampus in which most synaptic connections are preserved. In the hippocampal CA1 or CA3 areas, seizures can be induced by altering the ionic composition of the aCSF, e.g. lowering calcium ions concentration [26], removing Mg^2+^ [27] or by blocking GABA_A_ receptors [28]. In addition to these chemical approaches, electrical stimulation [29] using various patterns of stimulation can also be used to evoke seizures. One such protocol is the stimulus train-induced bursts (STIBs; [17], [29], [30]).

*In vivo* seizure on the other hand can be induced in rodents using chemicals [22], [31]–[34] or electrical stimulations [21], [22], [35]. Some of these models are believed to reflect epileptogenesis and are therefore useful in the search for antiepileptic drugs which may also prevent epileptogenesis [36]. Although finding such a drug appears to be a monumental challenge and elusive now (see [13]), concerted and sustained efforts in the development of new molecular entities with antiseizure activities is the likely way forward to eventually discovering agents that can abort the ictal process as well as prevent epileptogenesis.

Enaminones are a class of compounds that have been reported to possess anticonvulsant activity [15]–[17], [19], [37]. We and others have reported that these compounds modify GABAergic transmission as well as inhibit tetrodotoxin-sensitive sodium channels to produce effects on neuronal excitation that are consistent with anticonvulsant activity [16], [17], [19]. In this study, we have further investigated two dihalogenated enaminones (AK6 and E249) which we recently reported to have similar potency but with superior efficacy [38] than any other compound in this class. We tested the hypothesis that AK6 and E249 would suppress *in vitro* (chemically- and electrically-induced) and *in vivo* (electrically induced) seizures in rats with minimal CNS side effects. We hereby report that the 2,4-dibromophenyl enaminones AK6 and E249 are effective in suppressing *in vitro* seizures in hippocampal slices as well as protecting rats against electrically-induced seizures at a level comparable to phenytoin but with potentially minimal CNS side effects.

## Materials and Methods

### 1: Synthesis of AK6 (Compound 25) and E249 (Compound 21)

**Figure 1:**
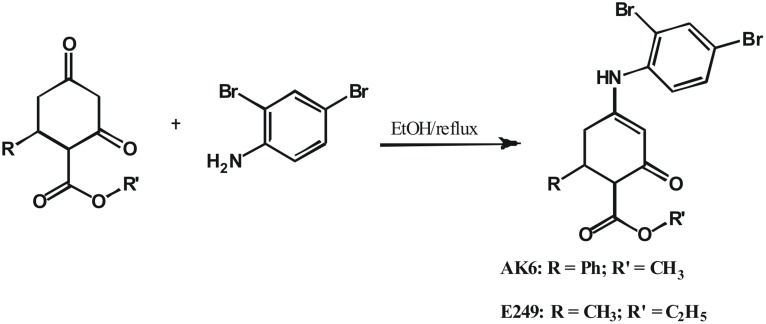
Summarized synthetic scheme for synthesis of AK6 (compound 25) and E249 (compound 21).

AK6 and E249 (figure 1) were synthesized for this study according to previously reported methods [15] with modifications to produce the desired analogs and to enhance yield [38]. These new analogs were thoroughly characterized using appropriate spectroscopic and analytical methods to confirm their structures.

### 2: Pharmacological Experiments

Male Sprague-Dawley rats (weighing 100–150 g) were used in all these experiments and were supplied by Kuwait University Animal Resource Centre. All experiments were done in accordance with guidelines for the care and use of experimental animals as contained in those established by the Canadian Council on Animal Care and was approved by the Kuwait University Health Sciences Center Animal Research Ethics committee. The procedures employed minimized animal suffering and the minimum number of animals necessary to produce the required results was used.

### 3: *In vitro* Seizures: Extracellular Electrophysiological Recording

For *in vitro* studies, field (extracellular) potential (population spikes:-PS) recordings were performed in coronal hippocampal slices generated from male Sprague Dawley rats (100–150 *g*) using previously published techniques and methods [16], [17]. Briefly, rats were deeply anesthetized with halothane and killed by quick decapitation. The brains were quickly removed and placed in ice cold (4°C) artificial cerebrospinal fluid (aCSF) that was bubbled continuously with 95% O_2_ and 5% CO_2_ (Carbogen). The composition of the aCSF used for dissection, storage and PS recording was (in mM) 120 NaCl, 3.3 KCl, 1.2 MgSO_4_, 1.3 CaCl_2_, 1.23 NaHPO_4_, 25 NaHCO_3_ and 10 D-glucose. Three hundred and fifty micrometers (350 µm) thick coronal slices of the forebrain containing the hip­pocampus were cut from a block of brain tissue in ice cold (4°C) aCSF using a Leica VT 1000S (Leica Microsystems, Wetzlar, Germany) tissue slicer. Prior to recording, slices were incubated for 1 h in aCSF which was continuously bubbled with carbogen at room tempera­ture (21–22°C). Slices were carefully trimmed of most cortical and midbrain tissue and suspended on a nylon mesh in a 500 µl capacity record­ing chamber. Bath temperature was tightly maintained at 29–31°C to ensure that changes in responses were not due to variation in temperature [39]. Slices were perfused at a flow rate of 2–3 mL/min with carbogenated aCSF. An extracellular field recording glass electrode filled with 3 M NaCl (tip resistance between 5–10 MΩ) was placed in the *stratum pyramidale* of area CA1 for recording and bipolar stimulating electrodes were placed in the *stratum radiatum* near area CA1 to activate Schaffer collateral/commissural fibers and single population spikes (PS) recorded.

Epileptiform multiple PS and spontaneously occurring epileptiform activity (spontaneous bursts: SB), were induced electrically and chemically. Electrically, a patterned high frequency stimulation was employed to induce afterdischarges (AD) and bursting- stimulus train-induced bursts (STIBs). Briefly, the high frequency stimulus trains consisted of 4 stimuli (10–30 V, 200 µµs) at 100 Hz repeated 15 times at 5 Hz and were applied every 4–5 min via the bipolar stimulating electrode until afterdischarges (ADs) occurred which were then often, but not always, followed by bursts. It often took about 5–10 repeats to get the primary ADs followed by bursts which occurred spontaneously (without stimulation). To promote ADs and STIBs occurrence, the aCSF was slightly modified by reducing the concentration of Mg^2+^ in the aCSF to 0.9 mM [17], [30], [40].

Chemically, epileptiform activity was induced by either enhancing NMDA receptor-mediated glutamate transmission or reducing GABA_A_ receptor-mediated GABA transmission. In the former, the amount of Mg^2+^ in the aCSF was completely eliminated (zero Mg^2+^ model) whilst in the latter, 100 µM picrotoxin was added to the aCSF and the KCl concentration boosted by 1.7 mM to a total extracellular K^+^ concentration of 5 mM [17], [30], [40]. Under both these conditions, the slices were then stimulated periodically to monitor for the development of multiple PS which is often accompanied by SBs even in the absence of afferent stimulation. On average, it took 30–60 minutes for multiple spikes and SBs to develop. Subsequent to this, all responses were monitored and shown to be stable for between 15–30 minutes prior to any drug application.

### 4: Data Acquisition, Analysis and Statistics

All PS recordings were made using an Axopatch 1D amplifier and pClamp software (Clampex 8, Molecular Devices, USA) in current clamp mode at sampling rates of 10 or 50 kHz, filtered at 1 kHz, digitized and stored for off-line analysis. Each stored trace was an average of 6 traces sampled at 10 second interval. The amplitude of the PS was measured from the peak of the positive going wave to the tip of the negative going wave. The number of spikes that occurred following afferent stimulation was counted while the number of SBs that occurred in one minute (recorded in gap-free mode) was counted and used to calculate the frequency of events (ADs or SBs). All drugs were perfused for 6 minutes except in the picrotoxin model where picrotoxin was applied for longer depending on the duration of experiment. All data are expressed as mean ± standard error (SE). Statistical significance of all measurements was determined using Student’s *t*-test (paired or unpaired where appropriate) and was considered significant at p≤0.05 using SigmaStat (Systat Software Inc., San Jose, CA, USA). PS amplitudes were normalized by taking the mean of 4–5 responses prior to drug application and dividing the rest of the values by this mean. These values were used for average time to effect plots and for drawing bar graphs. All the graphical representations were done using SigmaPlot (Systat Software Inc., San Jose, CA, USA), and CorelDraw (Corel Corp., Ottawa, Canada) software.

### 5: *In vivo* Experiments

*In vivo* seizure experiments were done using Ugo-Basile ECT Unit 57800 (Italy) equipped with ear clips for rats. Two models of electroconvulsive seizures were tested in this study; the 6 Hz model and the maximal electroshock (MES) model. For the 6 Hz model, seizures were induced in male rats (100–150 G) using a 40 mA current pulse, width 0.2 ms for total duration of 3 s adapted from a previously reported method [21]. Preliminary testing using different current strengths showed that this protocol consistently induced stage 4/5 seizures in rats. For MES, the protocol by [23] was slightly varied to suit the limitations of our equipment (maximum current output 100 mA). MES was induced using a 70 mA current pulse, 0.2 s duration at 60 Hz [41]. Preliminary experiments also showed that this protocol was consistent in inducing stage 4/5 seizures in the rats. All seizures were staged based on the classification reported by [42] see Table 1. Two trained experimenters observed each animal after the shock and both had to agree on the stage of a seizure for that animal to be staged. Rats were injected intraperitoneally (IP) with drug (AK6, E249, or phenytoin, all dissolved in dimethyl sulfoxide, DMSO) 30 minutes prior to testing with a shock. Control animals received equivalent volume of DMSO. Each test group consisted of a minimum 5 rats. A test rat was placed in a transparent Plexiglas cage with a transparent top cover and allowed to settle in the cage prior to clamping the ears with the ear clips. The programmed stimulation protocol was then applied by a single trigger of the trigger button. Throughout this study, stages 1 or 2 were taken as protection from seizure activity and stages 4 or 5 were taken as full seizures (unprotected). As a result of this scoring, each rat that was tested had an all or none score as reported in the results and figures.

**Table 1:** Staging of seizure in rats (Taken from: Models of Seizures & Epilepsy, Pitkanen et al, 2006).

Stages of seizure	Description
1	Mouth and facial movement
2	Head nodding
3	Fore limb clonus
4	Rearing
5	Rearing and falling:-tonic-clonic

Locomotor experiments were performed in similar age rats. Locomotor activity was monitored using an automated locomotor and behavior tracking system (Versamax 4.10, AccuScan Instruments Inc, Columbus, OH, USA) that employed gridded photocells in standardized activity cages (see detailed description by [43], [44]). On the first day of experiment, all animals were placed in the activity cage for 2 hours to acclimatize. Twenty four hours later, each animal was then injected IP with drug or solvent (DMSO) and the locomotor activity monitored for 2 hours. All experiments were done in batches of 4 as the system is a four cage setup. Digital horizontal activity in bins of 10 min were quantified over the 2-hour duration of each experiment. Total activity over this period was then obtained by summing the activities of all 12 bins.

### 6: Chemicals and Drugs

The enaminones, AK6 and E249 were synthesized in-house [38] and were dissolved in dimethyl sulfoxide (DMSO). Stock solutions of 10 mM were prepared, aliquoted and stored at −20°C and used within 3 weeks. Picrotoxin, DMSO and all salts used for the preparation of aCSF were obtained from Sigma-Aldrich Company (Steinheim, Germany). All drugs and chemicals were diluted with the appropriate aCSF to the final concentration and applied by bath perfusion. In all *in vitro* experiments, the drug was applied only after 15–30 min after acquisition to ensure that all responses were stable. Drugs were washed out (recovery) for 15–30 min with appropriate control aCSF not containing the tested drug.

## Results

We previously described the structure-activity-relationships of a series of di-halogenated phenyl enaminones by monitoring their effects on extracellularly recorded PS [38]. In this study, we also determined the mechanisms by which these new compounds acted to suppress neuronal excitation. Based on their suppression of PS amplitude and concentration-response characteristics, AK6 and E249 were adjudged most potent of this series and most efficacious of all enaminones tested to date. From this study, 10 µM was chosen to examine the actions of these two compounds on *in vitro* seizure models because it produced the most robust and consistent effect similar to those previously reported for another enaminone [16]. For *in vivo* seizure experiments, dose-response relationships against two models were performed and the best protective dose was selected for further central nervous system effect investigations.

### 1: AK6 and E249 Depress PS Amplitude Recorded in Area CA1 of the Hippocampus

**Figure 2:**
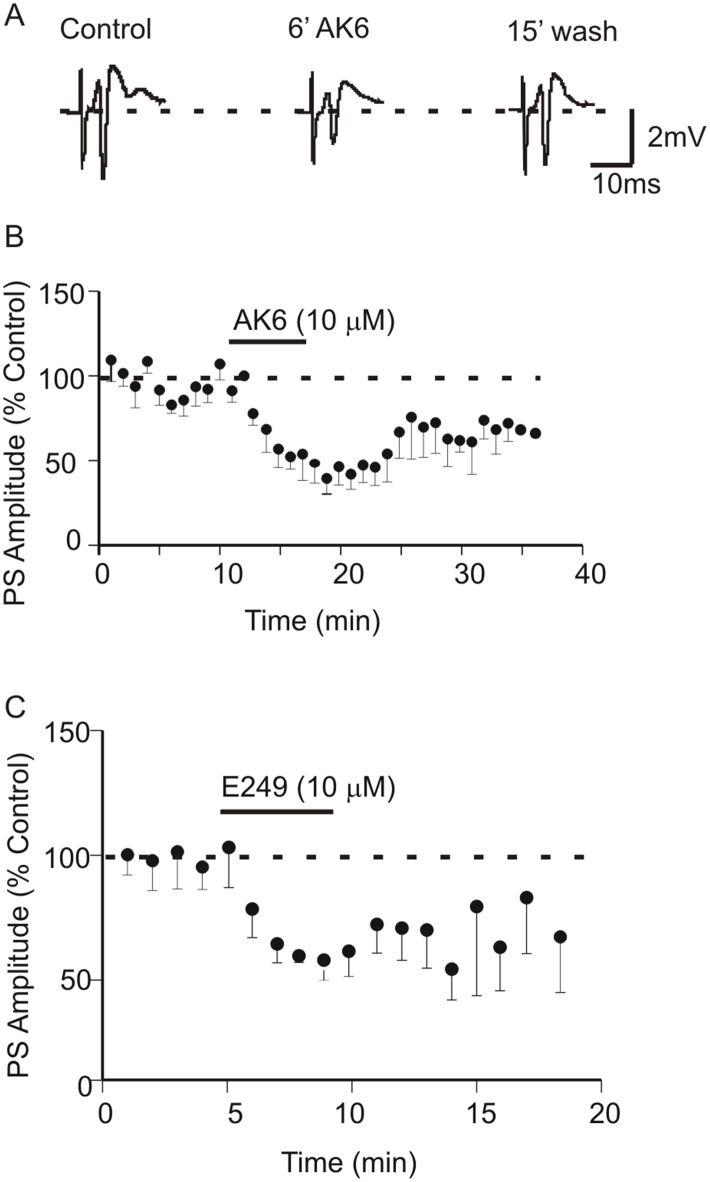
AK6 and E249 irreversibly depress population spikes (PS) recorded in the CA1 area of the rat hippocampal slice. A: Sample PS traces in control, after 6 minutes bath perfusion with 10 µM AK6 and following 15 min washout of the compound. B: Average time-effect plot of the effect of 10 µM AK6 on PS amplitude. C: Average time-effect plot of the effect of 10 µM E249 on PS amplitude.

As previously reported in [38] bath application of 10 µM AK6 produced consistent depression of the PS amplitude by −50.5±4.3% (p<0.05, paired *t*-test, n = 6, figure 2A & B). Maximal PS depression was observed after 6 min application and partial recovery (∼70%) occurred after 15 min washout of AK6. Similar to AK6, 10 µM E249 also depressed PS amplitude by −40.1±3.1% (p<0.05, paired *t*-test, n = 6, figure 2C) and this also showed a partial recovery of about 80% after 10 min washing out. Aspects of both these effects have been reported in the above study as part of the concentration-response curves.

### 2: AK6 and E249 Depress Chemically-induced Multiple Spikes and Spontaneous Bursts

**Figure 3:**
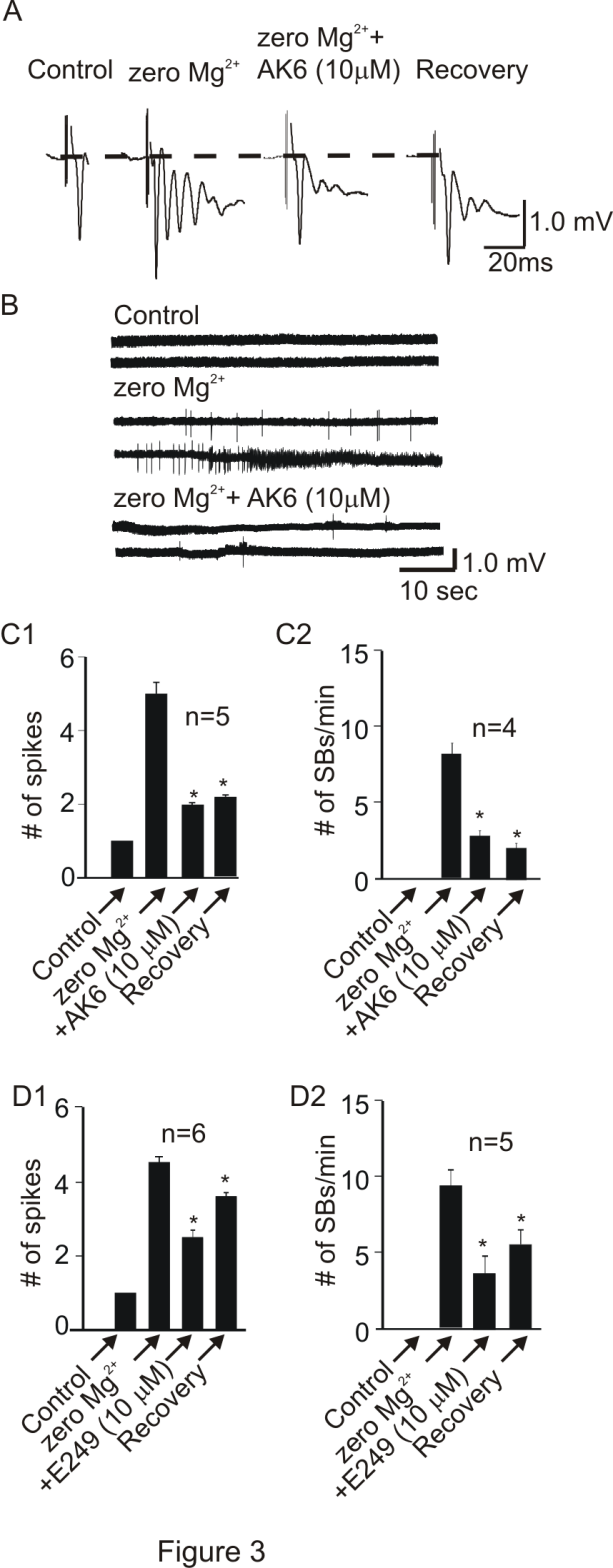
AK6 and E249 suppress zero Mg^2+^-induced epileptiform activity in the rat hippocampus. A: Sample traces of a single PS and multiple PS (mPS) induced by bath perfusion with aCSF in which magnesium was removed and effect of AK6 on mPS. B: Sample voltage traces (recorded in gap-free mode) showing that AK6 and E249 (only AK6 is shown) also suppress spontaneous bursts (SBs) frequency induced by the zero Mg^2+^ buffer. C1&2: Bar graphs summarizing the effects of AK6 on the number of spikes and SB frequency, respectively. D1&2: Bar graphs summarizing the effects of E249 on the number of spikes and SB frequency, respectively. In this and all other figures, * indicates statistical significance at p<0.05 compared to control.

In the first model, after recording an optimal PS, Mg^2+^ was removed from the perfusing buffer resulting in the unblocking of the voltage-dependent Mg^2+^ block of NMDA receptor [45] leading to the development of epileptiform activity in the slice [27], [46], [47]. After recording in zero Mg^2+^ buffer for about 20 min, a single PS was transformed into multiple PS (4.8±0.2, n = 11) in response to a single electrical stimulation of the afferents (figure 3A). AK6 reduced the number of multiple PS from 5.0±0.3 to 2.0±0.3 (–59.3±6.9%, p<0.05, paired *t*-test, n = 5, figure 3A & C). In addition to transforming a single PS to multiple PS, zero Mg^2+^ also induced SBs that occurred without stimulation. The SB frequency (7.8±1.9 bursts/min) was reduced by −65.9±7.2% (p<0.05, paired *t*-test, n = 4, figure 3B & C) to 2.6±0.9 bursts/min in the presence of 10 µM AK6. This inhibition did not recover after 20 min washout of AK6. Similar to AK6, 10 µM E249 also depressed the mPS by −48.8±9.5% by reducing the number from 4.6±0.3 to 2.4±0.5 (p<0.05, n = 6, paired t-test, figure 3D). Recovery from this effect was not substantial after 20 min wash. E249 also irreversibly depressed the SBs by −58.8±15.5% by reducing their frequency from 9.0±2.4 bursts/min to 3.9±1.2 bursts/min (p<0.05, paired *t*-test, n = 5, figure 3D).

**Figure 4:**
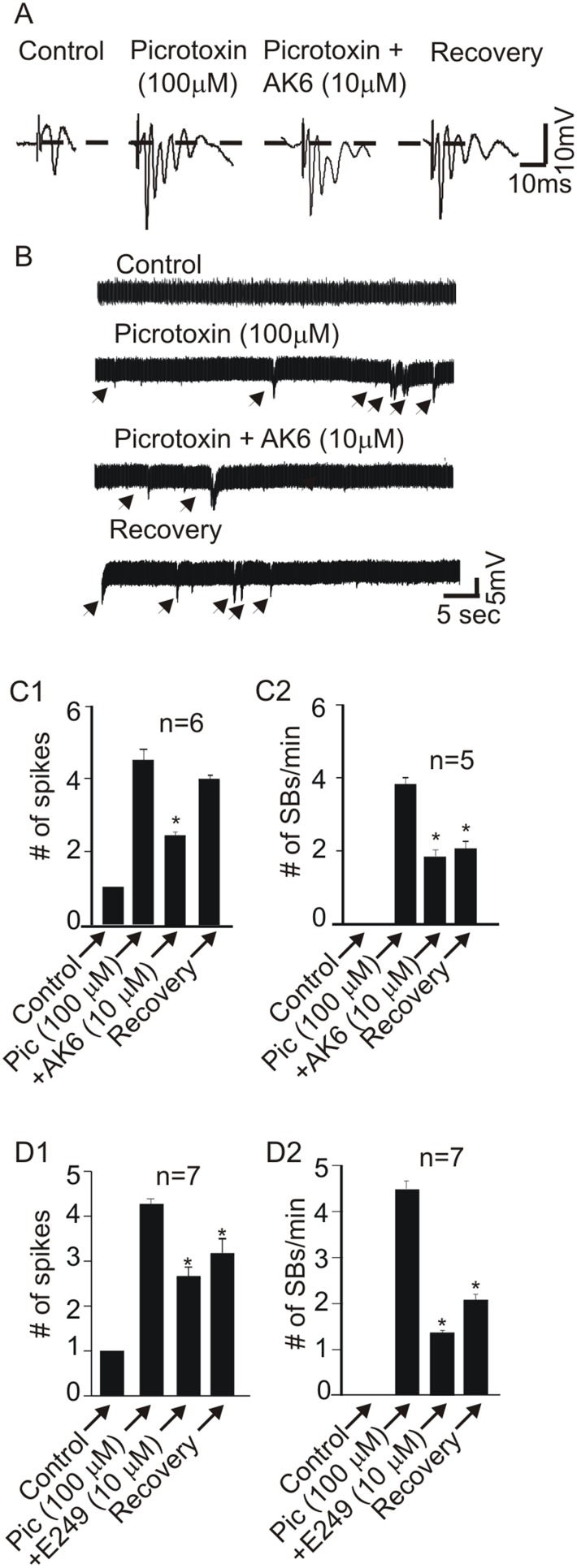
AK6 and E249 suppress picrotoxin induced epileptiform activity in the rat hippocampus. A: Sample traces of a single PS and multiple PS (mPS) induced by bath perfusion with aCSF containing 100 µM picrotoxin and the effect of 10 µM AK6 on the resulting mPS. B: Sample voltage traces (recorded in gap-free mode) showing that AK6 and E249 (only AK6 is shown) also suppress SBs (arrow heads) frequency induced by picrotoxin. C1&2: Bar graphs summarizing the effects of AK6 on the number of spikes and SB frequency, respectively. D1&2: Bar graphs summarizing the effects of E249 on the number of spikes and SB frequency, respectively.

In the second model of *in vitro* seizures, similar to the zero Mg^2+^ model above, picrotoxin (100 µM), a GABA_A_ receptor chloride channel blocker, was applied to slices for 20–30 min. This resulted in the removal of the strong inhibitory influence of GABA [48]–[50] on the CA1 neurons and the transformation of the single PS into multiple PS (4.4±0.1 spikes; n = 13, figure 4A) reflecting epileptiform activity in these pyramidal neurons. The amplitude and number of these spikes were allowed 15–30 min to stabilize. In 6 slices, the PS transformed into mPS with 4.5±0.1 spikes which were reduced to 2.5±0.2 spikes by 10 µM AK6 yielding a depression of −43.3±7.2% (p<0.05, paired *t*-test, figure 4A & C). Following 20–30 min washing, the number of spikes increased to 4.0±0.6 spikes yielding about 80% recovery. Similar to the zero Mg^2+^ model, mPS was also accompanied by SBs which occurred without stimulation. In the absence of AK6 (control), the frequency of SBs was 3.8±0.7 bursts/min which decreased to 1.8±0.2 bursts/min after 6 min application of 10 µM AK6. This represented a −50.0±8.3% depression (p<0.05, paired t-test, figure 4B & C) which recovered by only 10% after 20–30 min wash. Similar to AK6, 10 µM E249 also suppressed mPS number and SB frequency. In 7 slices, picrotoxin converted a single spike to 4.3±0.2 spikes which were reduced to 2.7±0.3 spikes (–37.1±5.5% depression, p<0.05, paired t-test, n = 7, figure 4D) after 6 min application. In these same slices, SBs frequency under control conditions was 4.6±1.8 bursts/min which was reduced to 1.4±0.3 bursts/min representing a suppression of −56.5±11.4% (p<0.05, paired t-test, n = 7, figure 4C). This effect also did not show appreciable recovery (about 15%) after 20–30 min wash.

### 3: AK6 and E249 Depress Electrically-induced Epileptiform Activity in Area CA1 of the Hippocampus

After recording a stable PS, application of several HFS (see methods) to Schaffer collateral/commissural fiber afferents to the CA1 pyramidal cells of the hippocampus was followed by characteristic epileptiform discharges; an initial primary afterdischarge (AD) immediately following the train (quantified in the first 30–60 seconds post-train) and subsequent spontaneous bursts (STIBs) in the absence of HFS [29], [30], [40]. These HFS-induced spontaneous bursts often occurred after about 5–10 applications of the STIB protocols (see methods) to the slice. As in other studies, AD and STIB did not occur in some slices attempted and here we are reporting only those slices in which we could induce AD and STIBs.

**Figure 5:**
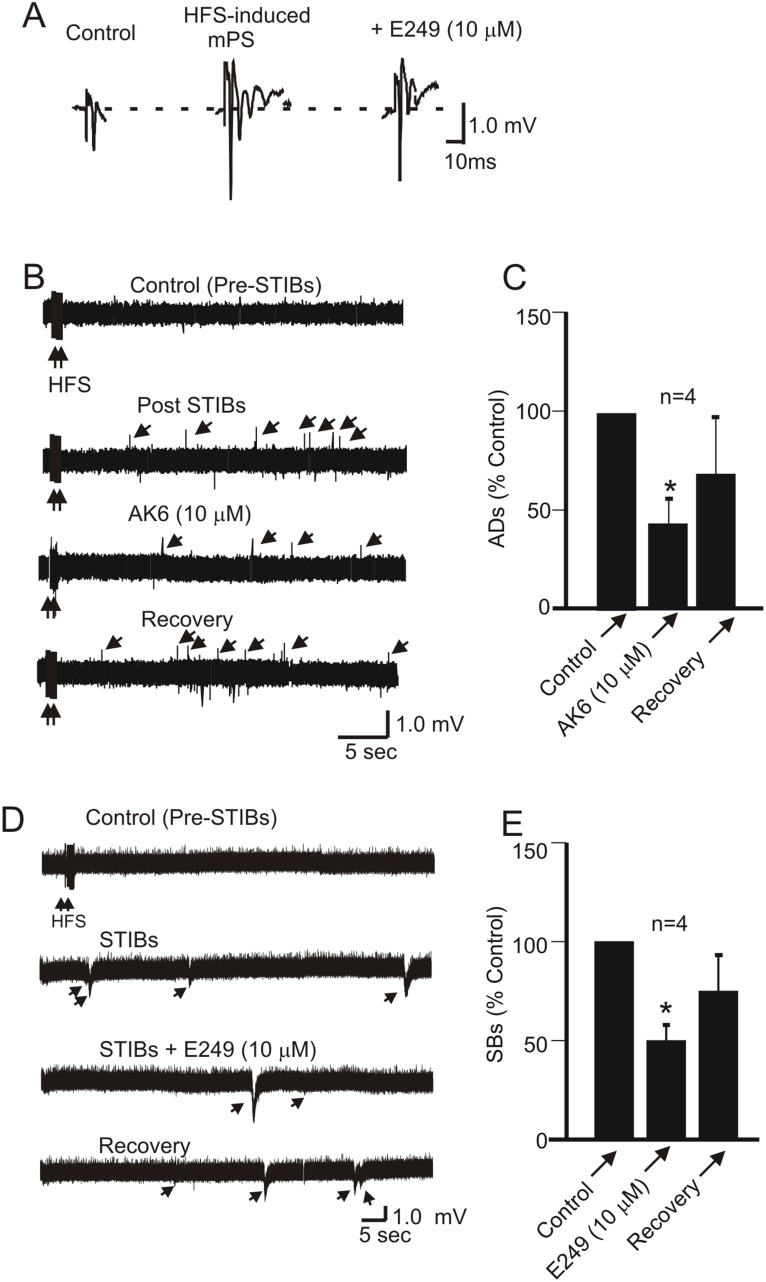
AK6 and E249 depress high frequency afferent stimulation (HFS)-induced epileptiform activity in the rat hippocampus. A: Sample traces of a single PS and multiple PS (mPS) induced by a patterned HFS (see methods). B: Sample voltage traces (recorded in gap-free mode) showing that AK6 suppresses afterdischarge (downward arrowheads) immediately following HFS. C: Bar graphs summarizing the effect of AK6 on the AD frequency. D: Sample voltage traces (recorded in gap-free mode) showing that E249 suppresses SBs, referred to as stimulus train-induced bursts (STIBs, upwards arrowheads). E: Bar graphs summarizing the effect of E249 on STIBs.

When epileptiform activity was successfully induced, the AD frequency was 9.8±1.9/min (n = 4) while the STIB frequency was 2.5±0.3/min (n = 4). AK6 at 10 µM depressed both the AD and STIB frequency by −57.9±16.4% (p<0.05, paired *t*-test, n = 4) and −66.7 (n = 1), respectively (figure 5 A–C). Similar to AK6, 10 µM E249 also depressed both the AD and STIB frequency by −38.6±3.4% and −50.0±7.9% (p<0.05, paired *t*-test n = 4), respectively (figure 5D & E). Both of these effects showed about 40% recovery after 20 min washing.

### 4: AK6 and E249 Protect Rats against Maximal Electroshock and 6 Hz Models of Seizures

**Figure 6:**
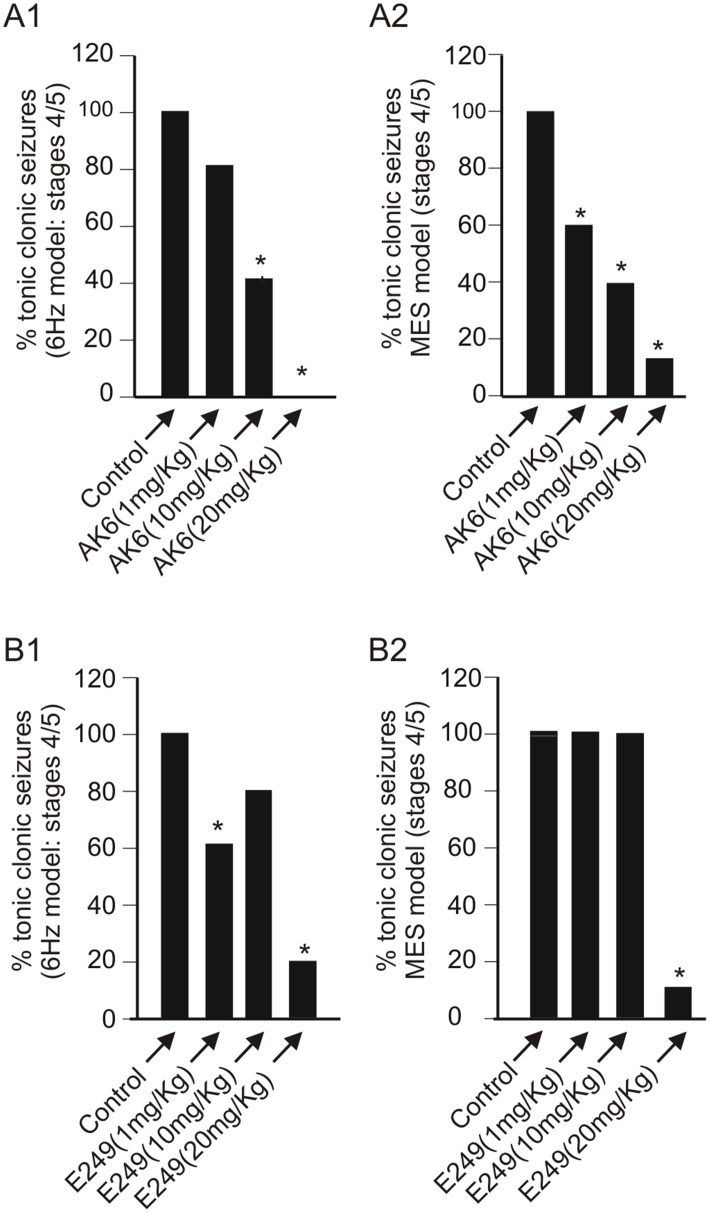
AK6 and E249 suppress maximal electroshock (MES) and 6 Hz seizures in a dose-dependent manner in male rats. A: Bar graphs summarizing the AK6 dose-dependent inhibition of seizures induced by the 6 Hz (A1, n = 20/5/5/8: control to highest dose of drug) and MES (A2, n = 18/5/5/8: control to highest dose of drug) models of seizures. B: Bar graphs summarizing the E249 dose-dependent inhibition of seizures induced by the 6 Hz (B1, n = 20/10/5/10: control to highest dose of drug) and MES (B2, n = 18/10/5/10: control to highest dose of drug) models of seizures. Note that the control bars and the 20 mg/kg bars for AK6 and E249 in this figure and those in figure 7 are the same.

**Figure 7:**
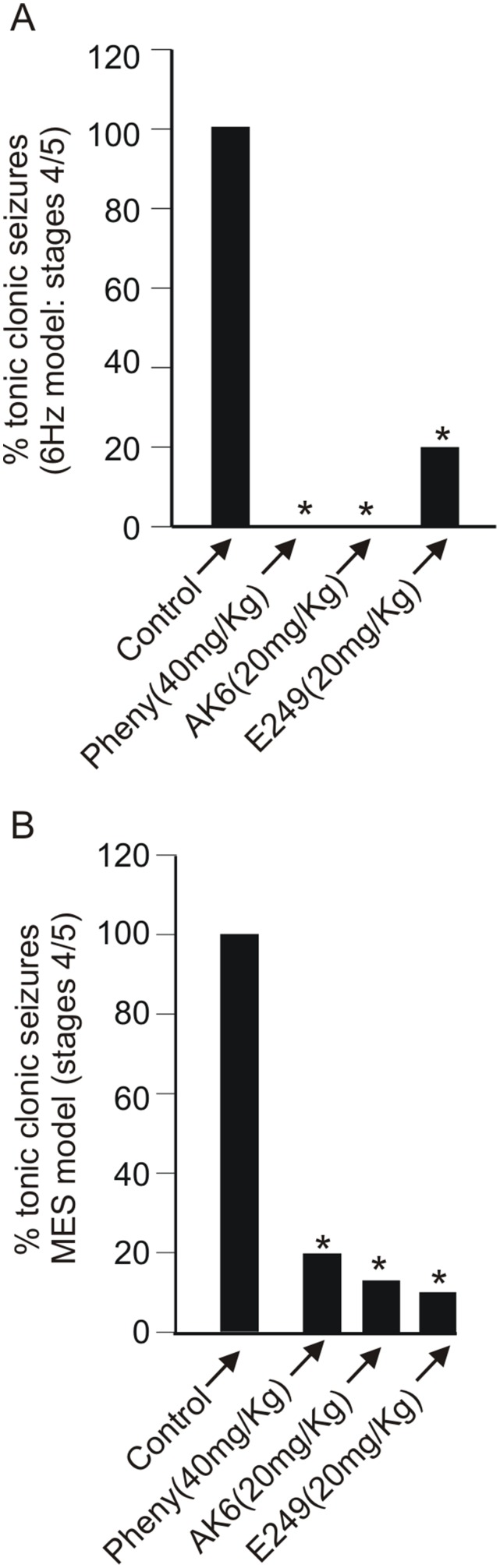
AK6 and E249 suppression of MES- and 6 Hz-induced seizures are comparable to those of phenytoin. Bar graphs summarizing the effect of AK6 (20 mg/kg), E249 (20 mg/kg) and phenytoin (40 mg/kg) on the 6 Hz (A, n = 5–20) and MES (B, n = 5–18) models of seizures.

To test the anticonvulsant or antiepileptic effects of these compounds, two *in vivo* seizure models were employed. The optimal current and frequency required to induce stage 4/5 seizures (see table 1) in both MES and 6 Hz models were determined in preliminary studies in 40 rats (see methods). In 20 control rats that received only saline injection, stages 4/5 seizures could be induced in all rats (100% response) to the optimal stimulation protocol used to induce seizures in the 6 Hz model. Different groups of rats (5–8 each) were then pretreated with AK6 at different doses of 1, 10 and 20 mg/kg for 30 min prior to subjecting them to the 6 Hz protocol. Rats were considered protected if they were scored by both observers as either stage 1 or 2 on the seizure scale. The level of protection, which was a reduction in seizure activity from stages 4 or 5 to stages 1 or 2, was 20% (1 out of 5 rats tested), 60% (3 out of 5 rats tested) and 100% (8 out of 8 rats tested), respectively compared to 0% in control (0 out of20 rats tested; figure 6A) yielding a calculated ED_50_ of 9.9 mg/kg. In another series of experiments using the MES protocol, AK6 pretreatment also protected rats in a dose-dependent manner whereby 1, 10 and 20 mg/kg protected by 40% (2 out of 5), 60% (3 out of 5) and 87.5% (7 out of 8), respectively compared to 0% protection in control 0 out of 15; figure 6A) with a calculated ED_50_ of 9.8 mg/kg. However, when similar experiments were repeated for E249, a less dose-dependent protection was observed on both 6 Hz and MES models whereby only 20 mg/kg produced a significant protection of 80% (8 out of 10) and ∼90% (7 out of 8) for 6 Hz and MES, respectively (figure 6B). Although 1 mg/kg appeared to produce 40% protection in the 6 Hz model, this was likely an experimental aberration since 10 mg produced less protection. In these studies, because both drugs were dissolved in DMSO, further experiments were done whereby rats (5 per protocol) were injected IP with equivalent volumes of DMSO and this did not protect any rats from seizures induced by any of these protocols. As DMSO produced identical results as dry run and saline injected animals, these animals were all combined as control in figures 6 & 7.

In the above studies, 20 mg/kg of both AK6 and E249 was clearly the most effective dose in protecting rats against both 6 Hz-induced and MES-induced seizures. This dose was chosen for comparative studies with the prototypical antiepileptic drug phenytoin on another set of rats (figure 7; note that in this figure, the effects of AK6 and E249 at 20 mg/kg are taken from the dose response studies above). The dose of phenytoin reported to be effective in similar studies was 40 mg/kg hence we employed this dose in these comparative experiments [21], [51]. Phenytoin (40 mg/kg; 5 rats) and AK6 (20 m/kg; 8 rats) protected all rats from seizures induced by the 6 Hz protocol while E249 (20 mg/kg, 10 rats) protected 80% (8 out of 10) of rats. In the MES protocol on the other hand, E249 provided 90% protection (9 out of 10), phenytoin (40 mg/kg) provided 80% (4 out of 5) and AK6 (20 mg/kg) protected 87.5% of the rats (7 out of 8).

### 5: AK6 does not Affect Locomotor Activity in Rats

**Figure 8:**
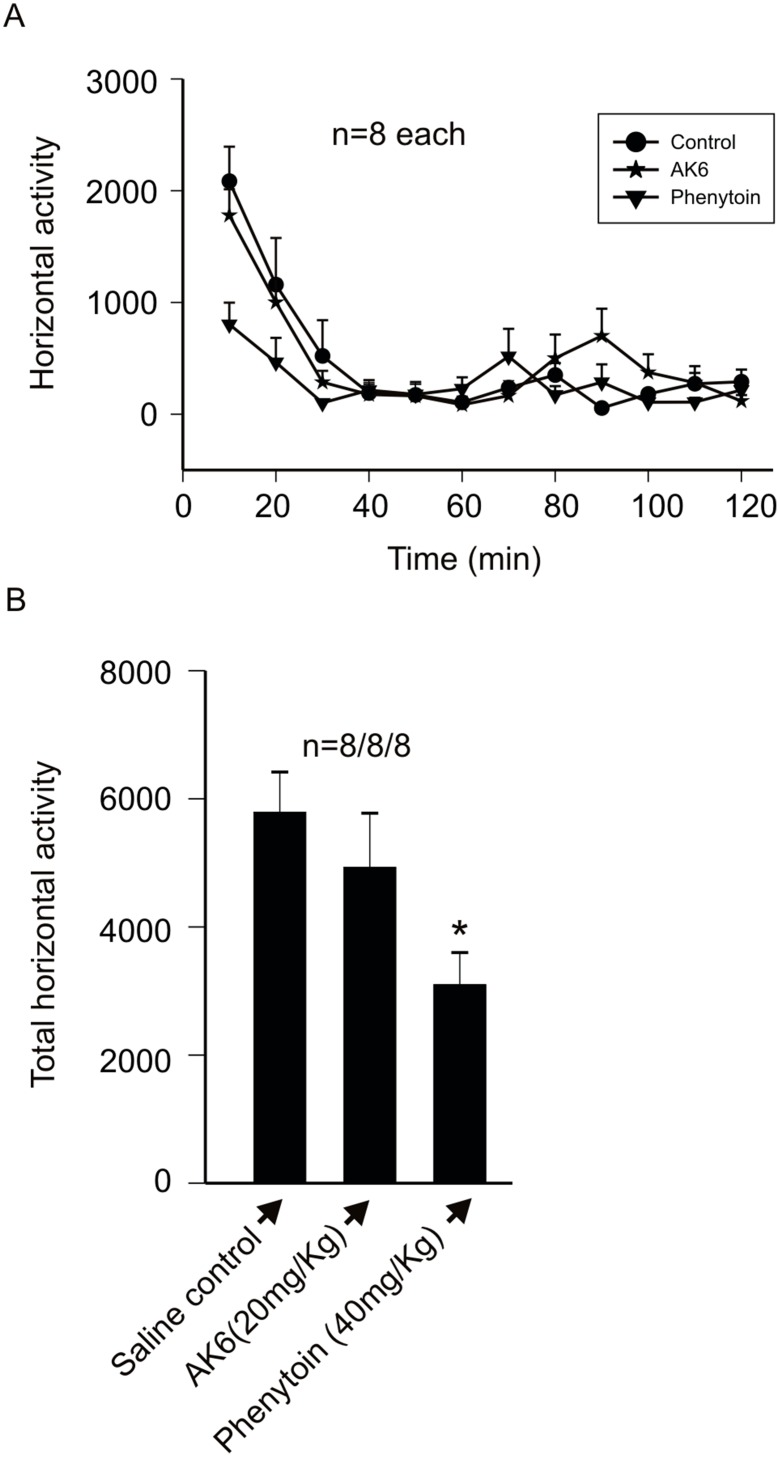
AK6 has no effect on locomotor activity in male rats. A: A time to effect plot of the horizontal motor activity of rats in control (saline injection), after AK6 (20 mg/kg) and after phenytoin (40 mg/kg) all given IP. B: Bar graph summarizing total horizontal locomotor activity (2 hours) of all rats in A.

One of the main drawbacks of currently available antiepileptic drugs is the occurrence of central nervous system side effects such as somnolence, drowsiness and ataxia [52], [53]. As all these effects will be expected to decrease locomotion, we tested if AK6, the most efficacious, had effects on locomotion in rats and compared these to those of phenytoin. Using the automated locomotion tracking device, groups of rats were injected with saline, 20 mg/kg AK6 and 40 mg/kg phenytoin and their locomotor activity monitored over 2 hours. As shown in figure 8A, 10 minutes after IP injection of phenytoin there was already significant decrease in locomotion in this group of rats while AK6 was comparable to the saline injected group. When the total horizontal activity over the 2 hours period was calculated, control rats had a value of 5786.3±632 (n = 8), AK6 had 4925±848 (p>0.05 compared to control, n = 8) and phenytoin 3095±491 (p<0.05 compared to control, n = 8; figure 8B).

## Discussion and Conclusions

This study is an extension of our recent reported structure-activity relationship studies of dihalogenated enaminones where the dibromophenyl analogs were found to have superior activity in suppressing neuronal excitation [38]. The anticonvulsant potential of two of these compounds, AK6 (compound 25) and E249 (compound 21), that showed the highest efficacy of all tested enaminones have now been investigated on *in vitro* and *in vivo* seizure models. We show in this study that, at the optimal concentration of 10 µM, AK6 suppressed chemically and electrically induced multiple spiking by 45–65%, an effect that lasted beyond the 15–20 min washout period employed throughout the study. The relatively less efficacious analog, E249 at the same concentration also suppressed these epileptiform events by about 40–55%. Both these analogs protected rats against two electrically-induced seizure models, MES and 6 Hz, in a dose-dependent manner. At the highest dose tested (20 mg/kg which is equivalent to 4X *in vitro* concentration of 10 µM≡4.8 mg/kg) both AK6 and E249 produced protection that was equivalent to that produced by 40 mg/kg phenytoin, an anticonvulsant agent that has been in clinical use for a very long time. By contrast to phenytoin which suppressed locomotor activity in rats by nearly 50%, 20 mg/kg AK6 had no significant effect on locomotion recorded in rats.

Our results show that AK6 and E249 are effective in depressing both chemically- and electrically-induced seizures *in vitro*, which is strongly supported by their actions *in vivo* in depressing MES and 6 Hz seizures. Similar to our previous findings that E139, a bromophenyl enaminone had action at both the synaptic level [16] involving GABA and on the postsynaptic cell body [54], we also observed that the dibromophenyl analogs worked by similar mechanisms affecting both synaptic and postsynaptic cell properties of neurons with similar potency but better efficacy [16], [17], [38].

The ability of AK6 and E249 to depress the number of spikes induced by both chemical (picrotoxin and zero Mg^2+^ buffer) and electrical seizure protocols suggests that these enaminones have broad based anti-seizure properties. Both compounds also markedly suppressed the occurrence of spontaneous bursts that usually accompany the multiple spiking described above. The multiple spikes following afferent stimulation are generally taken to represent ictal events while the spontaneous bursts are thought to correspond to interictal events *in vivo* [27]. The interictal events may then summate or coincide with other synaptic events or action potentials leading to the aberrant activity that is characteristic of the ictal period [14], [55]–[59]. Although AK6 was superior to E139 (a monobromo analog of AK6 having a 6-methyl substituent on the cyclohex-3-ene ring) in depressing population spike amplitude (magnitude and duration), its ability to suppress multiple spikes and spontaneous bursts was not superior to E139 at equivalent concentrations in both chemically- and electrically-induced seizure models [17]. This suggests that, while these dibromo analogs in this study may provide a similar level of protection against seizures, they may do so at a lower dose which may then afford lesser or fewer side effects.

We had hypothesized in our earlier publication on E139 [17] that enaminones may have antiepileptogenic activity because of their actions on spontaneous bursts. Pretreatment with E139 however did not prevent the development of epileptiform activity in slices. As this series of compounds appear to produce their effects using the same or similar mechanisms as E139, it is unlikely that they would prevent epileptiform activity when pre-exposed since E139 did not.

Both AK6 and E249 significantly protected rats from two *in vivo* seizure models. Both the maximal electroshock (MES; used to present generalized seizures) and 6 Hz model (thought to represent partial psychomotor seizures) have been used among others for preclinical screening of potential anticonvulsant agents or compounds [35], [60], [61]. In these *in vivo* studies, AK6 at the dose of 10 mg/kg (equivalent to ∼twice the *in vitro* concentration used:-10 µM = 4.8 mg/kg) protected more than 50% of rats from both MES and 6 Hz seizures while 20 mg/kg protected 100% of rats tested. E249 on the other hand was effective in protecting rats in both models only at 20 mg/kg but the suppression at this dose was quite robust. By comparison to phenytoin (40 mg/kg), both E249 and AK6 at 20 mg/kg showed comparable abilities to protect rats against both MES and 6 Hz seizures. These *in vivo* effectiveness of AK6 and E249 on MES and 6 Hz is in contrast to other GABAmimetic agents (e.g. tiagabine) which have been reported to be ineffective against MES [62]. This can be explained by the observation that enaminones may cause antiseizure activity by more than one mechanism of action [16], [17].

One of the biggest challenges in epilepsy pharmacotherapy today is the common occurrence of CNS related side effects [6], [7], [63] in particular ataxia and sedation. Using locomotor activity as an index of ataxia and possibly sedation, we compared the effect of phenytoin with that of AK6. At the equivalent doses that produced similar protection against MES- and 6 Hz-induced seizures, phenytoin significantly depressed locomotor activity in rats while AK6 had little to no effect on locomotion. This suggests that while producing comparable level of protection with phenytoin, AK6 may have a superior safety profile. In addition to their demonstrated effectiveness in MES and 6 Hz seizure models, for better clinical relevance and application, we need also to investigate the ability of AK6 and E249 to protect against chronic seizure models such as amygdala and hippocampal kindling models [35], [64]. Furthermore, pharmacokinetic and enzyme induction studies [65] are required to fully characterize these new and promising anticonvulsant compounds and to determine if they possess potential for further development into clinical trials.

In conclusion, the dibromophenyl enaminones AK6 and E249 depress *in vitro* and *in vivo* seizures and AK6 has no effect on locomotor activity in rats. These compounds have similar effects on neuronal activity and on *in vitro* seizures as the previously reported E139 but with better efficacy. They also have *in vivo* anticonvulsant activity similar to phenytoin, a clinically available antiepileptic drug. However, AK6 (and likely E249) appear to be devoid of CNS side effects commonly associated with most anticonvulsant agents currently in clinical practice.
